# Functional and radiological outcomes after treatment of congenital pseudarthrosis of the tibia using the Ilizarov technique: a retrospective single-center study

**DOI:** 10.1186/s10195-022-00667-2

**Published:** 2022-09-23

**Authors:** Ahmed Ibrahim Zayda, Mohamed Kamal Mesregah, Soliman Hassan Zalalo, Samy Abdel-Hady Sakr

**Affiliations:** Department of Orthopaedic Surgery, Faculty of Medicine, Menoufia University, Menoufia, Shebin-El-Kom, Egypt

**Keywords:** Congenital pseudarthrosis, Ilizarov fixator, Intramedullary rod, Union rate, Refracture, Residual complications

## Abstract

**Background:**

Congenital pseudarthrosis of the tibia (CPT) is a challenging problem in orthopedic practice, with high rates of non-union, refracture, and residual deformities. After union, long-term follow-up is required to manage late post-union complications. This study aimed to assess the outcomes of the Ilizarov technique in the management of CPT.

**Materials and methods:**

This retrospective study included patients with CPT treated with the Ilizarov method between 2005 and 2018. Intramedullary rods were used in 9 cases and iliac bone graft was used in 12 cases. An orthosis was applied till the end of follow-up in all cases. The American Orthopaedic Foot and Ankle Society (AOFAS) scale was used for the evaluation of the functional outcomes.

**Results:**

This study included 16 patients, 11 males and 5 females, with an average age of 5.4 ± 2.8 years. Seven cases had multiple previous surgeries. Six patients had neurofibromatosis. The mean follow-up period was 5.8 ± 3.4 years. The average AOFAS score improved significantly from 47.5 ± 7.6 preoperatively to 78.9 ± 8.9 at the latest follow-up.

Union was achieved in 15 cases, and persistent non-union occurred in one case. The clinical results were excellent in one patient, good in seven cases, fair in 6, and poor in 2 cases. The radiological results were excellent in one patient, good in seven cases, fair in seven, and poor in one case.

**Conclusions:**

The Ilizarov technique combined with intramedullary rod and primary or secondary bone graft provides a high union rate of CPT and can achieve simultaneous effective management of problems related to pseudarthrosis, including non-union, deformity, limb shortening, and adjacent joint contracture and subluxation.

*Level of evidence* Level IV.

## Introduction

Congenital pseudarthrosis of the tibia (CPT), also known as congenital tibial dysplasia, is a rare condition with a reported incidence of between 1:140,000 and 1:250,000 live births [1].

CPT has variable and unpredictable outcomes and is known for non-union and refractures [2–4]. The etiology of CPT is unknown, although it was reported to be linked to neurofibromatosis-1 (NF-1) [2].

Anterolateral bowing of the tibia is usually the first sign of CPT, followed by secondary bowing of the fibula [5]. On radiographic examination, the tibia and fibula are broadened, with focal cortical sclerosis [6]. Fractures of the tibia and fibula can occur spontaneously or as a result of minor trauma. The subsequent bone healing is insufficient, resulting in pseudoarthrosis [4, 7].

The periosteum surrounding the pseudoarthrosis is thickened and thought to contribute largely to the development of the lesion [8, 9].

Patients in whom the fracture site has united often have a limb length discrepancy (LLD) and residual tibial and ankle deformities. As the deformity progresses, degenerative arthritis of the ankles and knees may develop [10, 11].

Besides achieving union and correcting deformities and LLD, the goal of treatment is also to ensure a functional limb with minimal interventions [2–4].

CPT can be surgically treated with different methods, including intramedullary rods or nails, vascularized fibular grafting, Ilizarov ring fixation, the cross-union method, or a combination of two or three techniques [3, 4, 12–14].

Surgery utilizes the same biological principles regardless of the option chosen, including pseudarthrosis excision, bone bridging of the defect, stable fixation, and angular deformity correction [15].

Making the choice of the ideal procedure is challenging, and it depends on various aspects, such as age, CPT type, outcomes of previous surgeries, and what constitutes a successful result [4, 16].

The Ilizarov method was popularized as a viable treatment option as it can address pseudarthrosis, LLD, and the associated complex multilevel and multidirectional deformities. Furthermore, it can be used in cases where other methods have failed [15, 16]. The overall reported union rate for the Ilizarov technique ranges from 60 to 100% [17].

The main principles of the Ilizarov treatment include meticulous resection of the pseudarthrosis, correction of angular deformities and joint orientation, stable fixation for healing, and length gain by distraction osteogenesis [15, 18, 19].

Few investigators have evaluated the functional results of these patients as they grow into adulthood. Although the incidence of refracture is as high as 47% [20], few published data are available regarding the incidence and management of refracture in adulthood.

In the current study, we aimed to evaluate the functional and radiological outcomes and the complications and their management following treatment of congenital pseudarthrosis of the tibia using the Ilizarov method.

## Materials and methods

This retrospective study included patients with congenital pseudarthrosis of the tibia (CPT) treated by segmental resection and the Ilizarov technique with a minimum follow-up period of 2 years. Surgeries were performed between 2005 and 2018 by a single orthopedic surgeon (A.I.Z.) who is highly experienced in pediatric deformity correction. Institutional review board (IRB) approval and consent from the parents of patients were obtained prior to conducting the study.

Inclusion criteria were patients with segmental tibial dysplasia with or without previous surgeries, aged 3 years or older, without ischemia or serious neurological deficits, and with or without associated deformities. We excluded patients under 3 years of age, those with impaired foot circulation, and those with serious posterior tibial nerve damage.

### Preoperative assessment

The affected limbs were examined for deformities, skin condition, neurovascular status, the site of the lesion, ranges of motion of the hip, knee, ankle, and foot joints, and motion at the site of pseudarthrosis. All patients were examined for leg length discrepancy and stigmata of neurofibromatosis. The type of CPT was categorized according to the El-Rosasy–Paley classification [7] for CPT. Patients were categorized into primary cases presented to us without prior surgeries or revision cases who had previous surgeries elsewhere.

### Surgical technique

Under general anesthesia and fluoroscopic guidance, surgeries were performed on a radiolucent table in a supine position. A tourniquet was used in all cases.

The pseudarthrosis site was approached anteriorly by incising the skin, subcutaneous tissue, and deep fascia opposite the diseased part of the tibia. The pseudarthrosis was then excised using a saw till healthy bone edges were reached, and the medulla was gradually opened by applying drill bits of increasing size and reamers (4–11 mm) that were suitable for the tibial size until a normal medullary canal was obtained. Great caution was used to not excise too much normal bone and to preserve the maximum available healthy bone in these patients with an already small-sized tibia. Removal of the entire surrounding periosteum was done. At this stage, the tourniquet was deflated and removed to accomplish hemostasis.

A retrograde intramedullary rod was inserted in nine cases with large residual bone defects of more than 4 cm after intraoperative excision of the CPT and applying some shortening.

The components of the applied Ilizarov frame varied according to the age of the patient and the length of the remaining available bone. In most cases, there were three levels of fixation, including the proximal tibia, the transported middle segment, and the distal tibial metaphysis. In some cases, an additional level of fixation was applied in the distal femur to correct or prevent joint contracture and subluxation, and another level was added below the ankle joint in cases with a very short remaining distal tibia. In only one patient, two tibial rings were applied proximal to the corticotomy site, one middle ring was applied in the transported segment, and two distal tibial rings were used.

Intraoperative acute leg shortening was done in some cases with smaller intraoperative bone defects to a safe distance of 3–4 cm, guided only by intraoperative monitoring of distal pulse and capillary circulation. This intentional shortening allowed primary bone grafting in two cases at our first surgical interference, while in 10 cases, a secondary bone graft was done at a later stage once the main tibial segments met at the docking site.

Primary iliac bone grafting at the docking site was done in two cases. Proximal tibial corticotomy was then done, with great care taken to resuture the periosteum after corticotomy to get the best chance of a good regenerate. Closure of the wound was then done in layers with or without a suction drain.

In cases with retained hardware, it was removed first, and then the completion of surgery was resumed as described.

In one patient with previous multiple surgeries and atrophic bone ends and non-union, there were genu recurvatum and posterior subluxation of the knee. Therefore, the knee subluxation was first corrected by anterior translation of the tibia using a translation mechanism within a few days following surgery, regardless of the initiation of distraction at the corticotomy site 1 week postoperatively. A trial of the gradual correction of the genu recurvatum through the site of the regenerate was executed in the distraction phase and before full consolidation of the regenerate.

In two patients with sclerotic-type CPT, the fibula was hypertrophied and deformed, so segmental excision was done to correct leg alignment and facilitate leg lengthening.

In the single patient with a normal fibula, we kept it intact and only did tibial segment transport.

In patients with an atrophic but intact fibula, segmental excision was done, while in patients with an atrophic fibula with pseudarthrosis, there was no need for any special maneuver.

Great care to increase the diameter at the tibial docking site was taken, either by bone graft in our first surgery or weeks later, when the main tibial segments met. Bone graft was indicated if the cross-sectional diameter at the docking site was narrow, and it was also used to fill small residual defects after the main tibial segments met at the docking site.

In some patients who still had a small narrow tibia despite adequate debridement, multiple longitudinal osteotomies were done at the end of the proximal main tibia at the docking site, with the cross diameter of this tibial end enlarged by the gentle spreading of these longitudinal bone chips across the longitudinal osteotomies while they were still enclosed in their surrounding healthy periosteum (if possible).

### Postoperative care

On the first postoperative day, anteroposterior and lateral radiographs from the knee to the ankle joints were taken. Meticulous care was taken of the fixator pin sites.

The latent period was between 5 and 7 days postoperatively. Then we always started with a 1 mm per day rate, with a rhythm of 0.25 mm every 6 h. This rate and rhythm continued for 3–4 weeks and was sometimes modified later to a lesser extent according to the shape and quality of the regenerate.

Postoperative lengthening was done in two cases in which we achieved complete closure of the docking site and did a primary bone graft. In the remaining 14 cases, middle segment transport was done until it reached the docking site, and secondary bone grafting was done in 10 cases. Then we continued the lengthening to the targeted length.

X-rays were obtained every 2 weeks for 2 months, and then every month till the removal of the fixator.

Weight-bearing was allowed when tolerated, and the frame was removed after full solid union and the maturation of at least three cortices of the regenerate.

After removal, transcalcaneal wire was applied for an additional 1 or 2 months in four cases. In another five cases, a single intramedullary rod replaced the fixator to guard against refracture, and was exchanged periodically one to three times at intervals of 6–10 months in three cases according to tibial growth. The duration of use of the IM rod after frame removal in these cases ranged from 6 to 24 months.

After frame removal, a below-knee cast was applied for 1–2 months, which was then replaced by a leg–ankle–foot orthosis till the end of follow-up. During the follow-up period, union, refracture, progression of deformities after full union, and our interference were all reported.

### Methods of evaluation

The American Orthopaedic Foot and Ankle Society (AOFAS) scale [21, 22] was used for functional assessment.

Radiographic and clinical results were classified into excellent, good, fair, and poor using the criteria previously reported in the study by Inan et al. [23]; see Table 1.

**Table 1:** Classification of clinical and radiological results according to the study by Inan et al. [22]

Clinical results	
Excellent	Unrestricted activity
	Contact sports
	No LLD
	No pain
	No restriction of ankle and subtalar motion
Good	Noncontact sports
	LLD 2.5–4 cm
	Shoe modification compensates for limping
	Mild pain
	Slight restriction of ankle and subtalar motion
Fair	No sports
	LLD 4—5 cm
	Moderate pain
	Restriction of ankle and subtalar motion
Poor	Crutches for walking
	LLD > 5 cm
	Severe restriction or no ankle and subtalar motion

Radiological results	
Excellent	Radiographic union of pseudoarthrosis
	No axial malalignment in sagittal and coronal planes
	No ankle valgus
	No deformity in calcaneus > 30 degrees
	No LLD
Good	Healed pseudoarthrosis
	Axial malalignment 1–7 degrees in coronal or sagittal plane
	Valgus 1–5 degrees
	Calcaneal pitch (30–60 degrees)
	LLD 2.5–4 cm
Fair	Axial malalignment > 7 degrees in coronal or sagittal plane
	Ankle valgus > 8 degrees
	Calcaneal pitch > 60 degrees
	LLD 4–5 cm
Poor	Persistent pseudoarthrosis

*LLD* limb length discrepancy

The Paley classification [24] for pin-site problems was used to evaluate and guide the treatment of pin tract issues.

Valgus deformity at the ankle was graded according to the Malhotra classification [25].

The residual pain was described as mild (pain after sports activity), moderate (pain during walking), or severe (pain at rest).

The satisfaction of patients and parents was evaluated using a questionnaire of five items measuring function, appearance, duration of treatment, how likely the patient would be to agree to undergo the procedure again, and overall satisfaction, with scores ranging from + 2 (the best) to − 2 (the worst) [26].

### Statistical analysis

Data were analyzed using the IBM SPSS software package, version 20.0 (IBM Corp., Armonk, NY).

Categorical data were represented as numbers and percentages. The chi-square test was applied to investigate the association between the categorical variables.

Quantitative data were expressed as a range (minimum and maximum), mean, standard deviation, and median. The Wilcoxon signed-rank test for abnormally distributed quantitative variables was used to compare two periods. The Mann–Whitney test was used to compare two groups based on non-normally-distributed quantitative variables. The significance of the obtained results was judged at the 5% level. A* P*-value of < 0.05 was considered significant.

## Results

### Demographics and baseline characteristics

This study included 16 patients: 11 (68.8%) males and five (31.2%) females. The mean age at the index surgery was 5.4 ± 2.8 (range, 3–14.5) years. The right leg was affected in nine (56.2%) patients, while the left was affected in seven (43.8%) patients. Nine (56.2%) patients had not been operated on for CPT before, while seven (43.8%) patients had one to seven previous failed surgeries. Time from the first surgery to our interference was 2.8 ± 2.0 (range, 1–6.5) years. Six (37.5%) patients had neurofibromatosis.

**Fig. 1:**
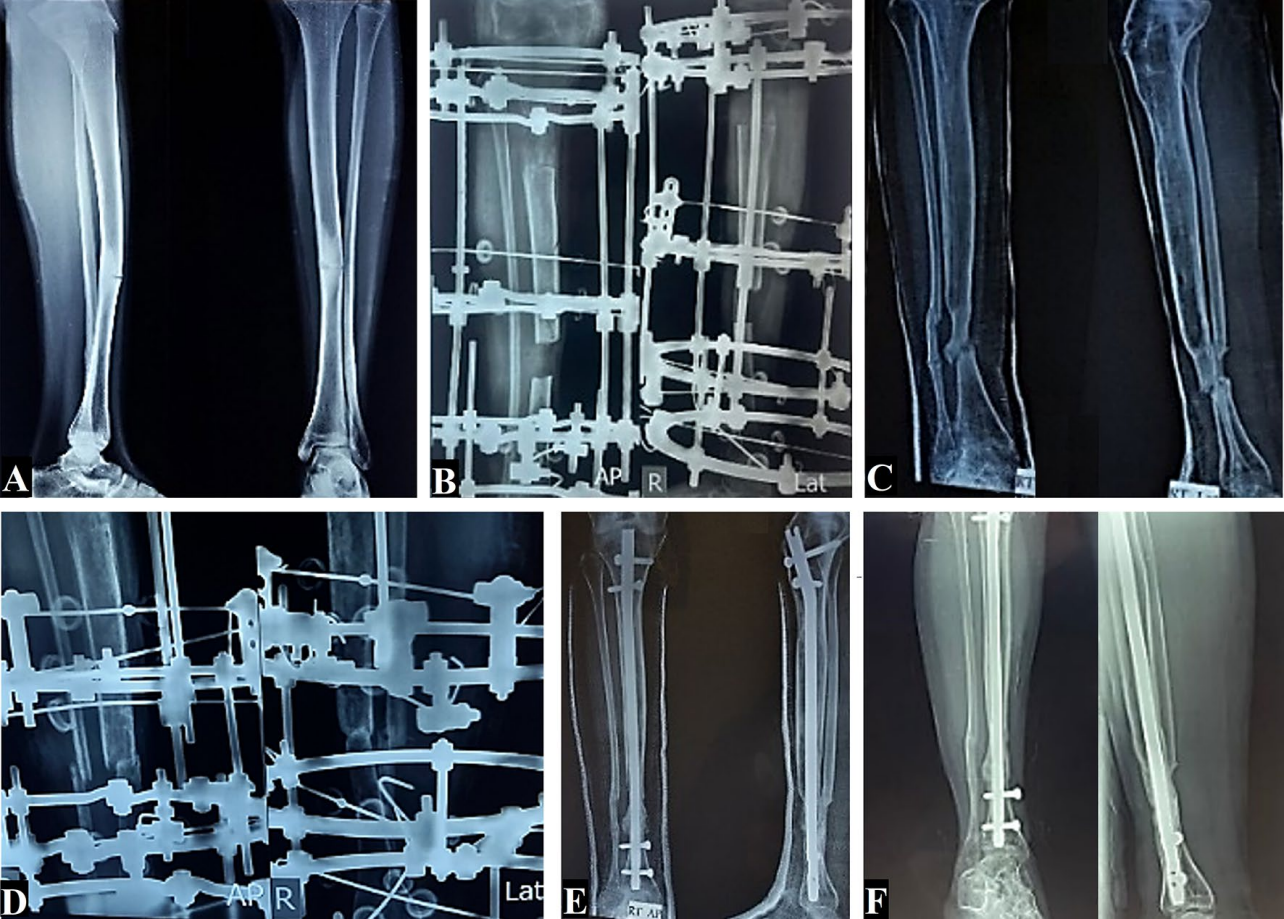
A 14.5-year-old female with congenital segmental tibial dysplasia without pseudarthrosis. **A** Preoperative AP and lateral X-rays. **B** Postoperative AP and lateral X-rays of Ilizarov segment transport after resection of the diseased segment. **C** Recurrence of sclerosis and narrow medulla with established pseudarthrosis 4 months post fixator removal. **D** Revision using another Ilizarov frame after limited debridement. **E** Maintenance of union by interlocking tibial nail. **F** AP and lateral X-rays showing complete union at 5.5 years’ follow-up

All patients had a displaced fracture with pseudoarthrosis except for one female patient, who had congenital segmental tibial dysplasia that did not progress into pseudoarthrosis until she had surgical treatment by segmental resection and segment transport by the Ilizarov method till union was achieved. Later, recurrence and refracture occured and the patient was managed successfully till solid union was achieved; see Fig. 1.

The average shortening of the affected limb was 3.6 ± 1.9 (range, 1–9) cm. According to the El-Rosasy–Paley classification of CPT, seven (43.8%) cases were type I, seven (43.8%) cases were type II, and two (12.5%) cases were type III. The fibula was dysplastic in 13 (81.3%) patients, hypertrophied in two (12.5%) patients, and normal in one (6.3%) patient.

The average preoperative gap at the CPT site was 1.3 ± 1.0 (range, 0.5–4) cm. After complete excision of the pseudoarthrosis, the mean gap was 6.1 ± 1.0 (range, 4.5–8.0) cm.

The mean preoperative AOFAS score was 47.5 ± 7.6 (range, 38–65); see Tables 2 and 3.

**Table 2:** Demographics and baseline characteristics

Characteristics	
Sex	
Males	11 (68.8%)
Females	5 (31.2%)
Age at index surgery (years)	
Mean ± SD	5.4 ± 2.8
Site	
Right	9 (56.2%)
Left	7 (43.8%)
Previous operation	
No	9 (56.2%)
Yes	7 (43.8%)
Time from 1st surgery (years)	
Mean ± SD	2.8 ± 2
Neurofibromatosis	
No	10 (62.5%)
Yes	6 (37.5%)
El-Rosasy–Paley classification	
Type I	7 (43.8%)
Type II	7 (43.8%)
Type III	2 (12.5%)
Fibula status	
Dysplastic	13 (81.3%)
Hypertrophied	2 (12.5%)
Normal	1 (6.3%)
Preoperative AOFAS score	
Mean ± SD	47.5 ± 7.6

**Table 3:** Details of the included patients

No./age (y)/sex	Side	Number of previous surgeries	Age at first surgery (y)	Type(s) of previous surgeries	Time from first surgery (y)	LLD (cm)	Preoperative bone defect (cm)	Intraoperative defect before shortening (cm)	CPT type	Preoperative AOFAS score	Index surgery (besides Ilizarov)	Additional surgeries	Duration of fixation (months)
1/5/M	Rt	2	3	Ilizarov	4	3	0.5	6	II	42	IM rod	Secondary BG	8
2/4.5/M	Rt	–	–	–	–	2	0.5	4.5	III	57	–	Exchange of wire	6
3/8/M	Rt	7	1.5	Ilizarov (*n* = 3) BG (*n* = 2) IM rod (*n* = 2)	6.5	9	3	8	II	57	IM rod	Exchange of wire	11
4/14.5/F	Rt	–	–	–	–	1	1	5.5	II	65	–	Secondary BG	8.5
5/3/M	Lt	1	1.5	Plate	1.5	4	2	5	II	48	Primary BG	Another BG then revision by 2 IM rods	7
6/5/M	Lt	1	3	IM rod	2	5	2	6	II	48	Primary BG	Exchange of wire and frame adjustment	8
7/4/F	Rt	–	–	–	–	3	1	6	I	44	IM rod	Secondary BG	8
8/6/M	Rt	1	5	Plate	1	4	2	8	II	48	IM rod	Secondary BG	11.5
9/7/F	Lt	3	3.5	Ilizarov (*n* = 1) BG (*n* = 2)	3.5	6	4	7	II	42	IM rod	Secondary BG	10
10/5/M	Lt	1	4	Plate	1	4	0.5	5	III	57	–	Frame adjustment	9.5
11/3.5/M	Rt	–	–	–	–	2	0.5	6	I	44	IM rod	Secondary BG	8
12/3.5/F	Rt	–	–	–	–	2	0.5	6	I	42	IM rod	Secondary BG	8.5
13/4/M	Lt	–	–	–	–	3	1	7	I	42	IM rod	Secondary BG	9
14/5.5/M	Rt	–	–	–	–	4	1	7	I	38	IM rod	-	8.5
15/4.5/F	Lt	–	–	–	–	3	0.5	5	I	44	–	Secondary BG	8
16/4/M	Lt	–	–	–	–	3	1	6	I	42	–	Secondary BG	8.5

*CPT* congenital pseudarthrosis of the tibia; *BG* bone graft; *IM* intramedullary

### Functional outcomes

The mean follow-up period was 5.8 ± 3.4 (range, 2–15) years. The clinical results were excellent in one (6.3%) patient, good in seven (43.8%), fair in six (37.5%), and poor in two (12.5%) patients.

The mean AOFAS score improved at the latest follow-up to 78.9 ± 8.9 (range, 56 to 96), and this improvement was statistically significant, *P  *< 0.001. The mean follow-up AOFAS score was no different if the IM rod was or was not used: 78.7 ± 5.7 and 79.1 ± 12.5, respectively, *P* = 0.920.

Pain around the ankles was moderate in three cases and was absent in 13 cases.

Knee range of motion was restricted in four cases by about 30 degrees of flexion and a lag in extension of 10–15 degrees. Spontaneous ankle and subtalar joint fusion occurred in one case. Limited ranges of motion of the ankle and subtalar joints occurred in nine patients; four cases were type I, three cases were type II, and two cases were type III according to the El-Rosasy–Paley classification.

All cases were able to bear weight, and nine cases were able to do noncontact sports activity while protected in the orthosis. One case with a residual shortening of 10 cm used shoe elevation to enable walk without crutches.

At the end of the follow-up, both leg lengths were equalized in five (31.3%) patients. LLD was reduced from 3.6 ± 1.9 cm preoperatively to a postoperative mean of 1.5 ± 2.8 cm, and this was statistically significant, *P* < 0.001; see Table 4.

**Table 4:** Comparison between the values preoperatively and at last follow-up of the LLD and AOFAS score

	Preoperative	Last follow-up	*Z*	*P*
LLD (cm)				
Mean ± SD	3.6 ± 1.9	− 1.5 ± 2.8	3.469	** < 0.001**
Median (min–max)	3 (1–9)	− 1 (− 10 to 3)		
AOFAS score				
Mean ± SD	47.5 ± 7.6	79.8 ± 8.9	3.523	** < 0.001**
Median (min–max)	44 (38–65)	82.5 (56–96)		

*Z* is from the Wilcoxon signed rank test

*SD* standard deviation

Regarding the final appearance of the leg, eight (50%) patients were extremely satisfied, two (12.5%) patients were moderately satisfied, four (25%) patients were unsatisfied, and two (12.5%) were extremely unsatisfied.

Regarding the final result of union and the function of the leg, nine (56.3%) patients were extremely satisfied, two (12.5%) patients were moderately satisfied, four (25%) patients were unsatisfied, and one (6.3%) patient was extremely unsatisfied.

### Radiological outcomes

The radiological results were excellent in one (6.3%) patient, good in seven (43.8%) patients, fair in seven (43.8%) patients, and poor in one (6.3%) patient.

At the latest follow-up, the average calcaneal pitch angle was 31 (range, 20–59) degrees. It was > 30 degrees in six (37.5%) patients. The mean healing index (HI) was 42.5 (range, 36.4–57) days/cm.

The average duration of Ilizarov fixation was 8.6 ± 1.4 (range, 6–11.5) months.

Union was achieved in 15 (93.8%) patients; it was achieved without a bone graft in four (25%) patients and with a bone graft in 11 (68.8%) patients (Table 5).

**Table 5:** Summary of the outcomes

Outcomes	
Follow-up period (years)	
Mean ± SD	5.8 ± 3.4
Follow-up AOFAS score	
Mean ± SD	78.9 ± 8.9
Union	
Yes	15 (93.8%)
No	1 (6.3%)
Final LLD	
Mean ± SD	1.5 ± 2.8 cm
Grading the clinical results	
Excellent	1 (6.3%)
Good	7 (43.8%)
Fair	6 (37.5%)
Poor	2 (12.5%)
Grading the radiological results	
Excellent	1 (6.3%)
Good	7 (43.8%)
Fair	7 (43.8%)
Poor	1 (6.3%)

*LLD* limb length discrepancy

### Complications

At the time of fixator removal, the alignment of the tibia in all cases was anatomical or at least within the accepted range, but with increased growth of the recently united tibia year after year, gradual malalignment was observed.

Valgus deformity at the ankle was observed in 11 cases, with a range of 5–15 degrees. In all cases of valgus deformity at the ankle, fibular pseudarthrosis was a frequent association. According to the Malhotra classification, five cases were grade 0, four cases were grade I, three cases were grade II, and four cases were grade II.

Treatment of valgus deformity at the ankle was by temporary medial distal tibial hemiepiphysiodesis in three cases with a deformity of more than 15 degrees and by observation in the other cases. No trial of medial wedge osteotomy was done, as the patients and their families refused any maneuver that involved rebreaking their legs after they had already recovered from a very long history of pseudarthrosis and a previous inability to walk.

Procurvatum deformity of the tibia of between 7 and 20 degrees occurred in four patients, and recurvatum of 10 degrees occurred in one patient.

Combined deformities existed in eight (50%) cases.

Valgus deformity at the knee existed in four (25%) cases, ranging from 10–20 degrees, and was noticed at 1–3 years’ follow-up. In two of those cases, temporary medial proximal tibial hemiepiphysiodesis was done, and the staples were removed later. Later, varus deformity at the knee with depression of the medial knee joint space in the coronal plane and an inverted slope of the upper tibial articular surface in the sagittal plane was reported in one case at 15 years of follow-up.

Pin tract infections of different grades occurred in all patients and responded to treatment with oral antibiotics and repeated wound dressing, except in three (18.3%) patients, who had grade III pin infections in two pins. These pins were removed, their tracts were debrided, and new wires were reapplied at other sites.

Persistent non-union occurred in one patient (case no. 5), and all trials to achieve union failed. In this patient, bone graft and intramedullary titanium rods were applied twice, but non-union persisted. Infection and resorption of the bone graft complicated the two operations; the infection resolved at the end of treatment, but the fracture did not unite. A protective orthosis was applied while waiting for natural growth of the tibia to provide enough length of a distal bone segment to accommodate strong fixation hardware in a later surgery.

**Fig. 2:**
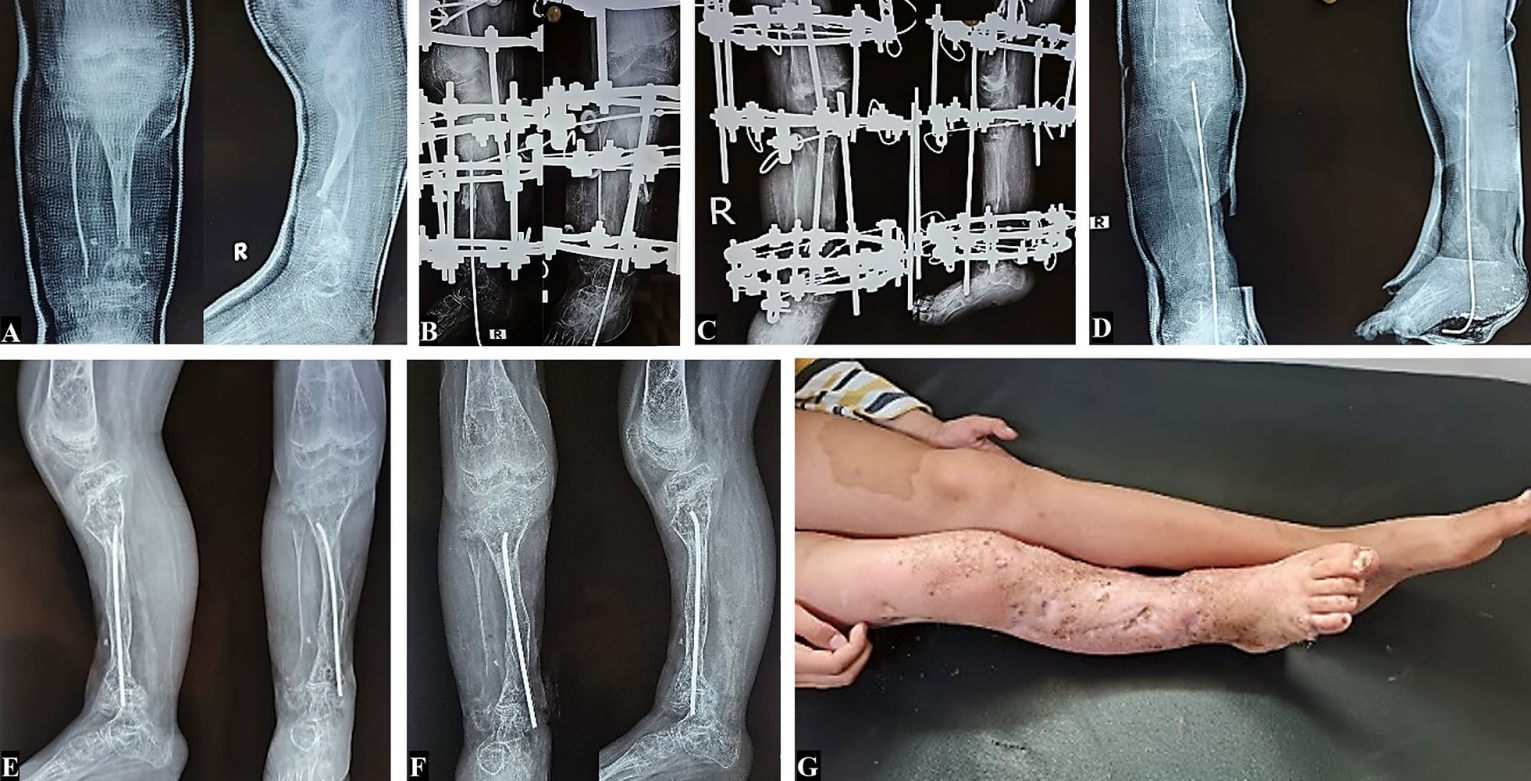
**A** Preoperative AP and lateral X-rays of a 7-year-old male who had undergone multiple previous surgeries for CPT since he was 1.5 years old, and had ended up with persistent CPT, posterior subluxation of the knee, genu recurvatum, and a very narrow, pencil-shaped tibia. **B** AP and lateral postoperative X-rays showing a trial correction of knee subluxation using a cross-knee Ilizarov frame, excision of the CPT, and compensation by proximal corticotomy. Note the relative widening of the end of the proximal tibia compared to the preoperative size. **C** Four months’ follow-up AP and lateral X-rays showing 6 cm distraction with a good regenerate. **D** AP and lateral X-rays taken immediately following fixator removal after full union with added transcalcaneal intramedullary K-wire for protection of the union. **E** 1.5 years’ follow-up AP and lateral X-rays showing complete union and consolidation of both the docking site and the regenerate, with widened tibia at the docking site and replacement of the K-wire by an IM titanium rod. **F** Two years' follow-up AP and lateral X-rays after changing the IM rod.** G** Clinical photograph taken at 2 years’ follow-up, showing 10 cm shortening of the affected limb

Ten cases had shortening of an average of 1.5 ± 2.8 (range, 1–10) cm; see Fig. 2.

**Fig. 3:**
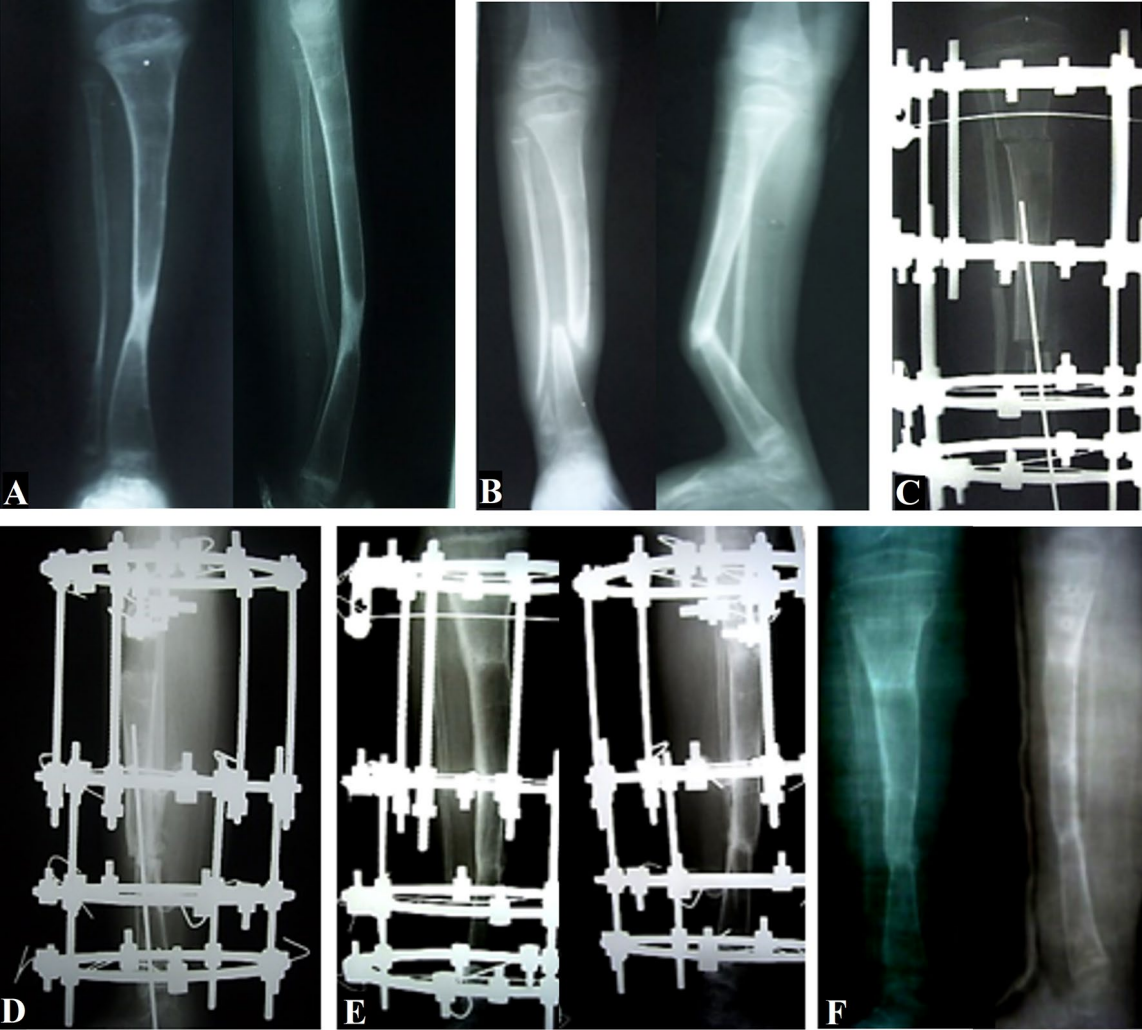
A case of a 4.5-year-old male who had undergone a previous excision of CPT when he was 3 years old. **A** AP and lateral X-rays showing recurrence of sclerosis and obliteration of medulla, and an impending fracture was noted. **B** AP and lateral X-rays showing refracture of the tibia and dysplastic fibula. **C** Immediate postoperative AP X-ray showing complete CPT excision, proximal corticotomy, intramedullary alignment using transcalcaneal wire, and near-total occlusion of the pseudarthrosis site by acute intraoperative shortening. **D** Lateral X-ray taken 8 weeks postoperatively, showing progression of union at the docking site and distraction at the regenerate. **E** Pre-removal AP and lateral X-rays showing complete union at the docking site and full consolidation of the regenerate. Note that the transcalcaneal wire was removed at an earlier stage. **F** AP and lateral X-rays taken 1 month after the removal of the frame, which show solid union without any sign of recurrence

**Fig. 4:**
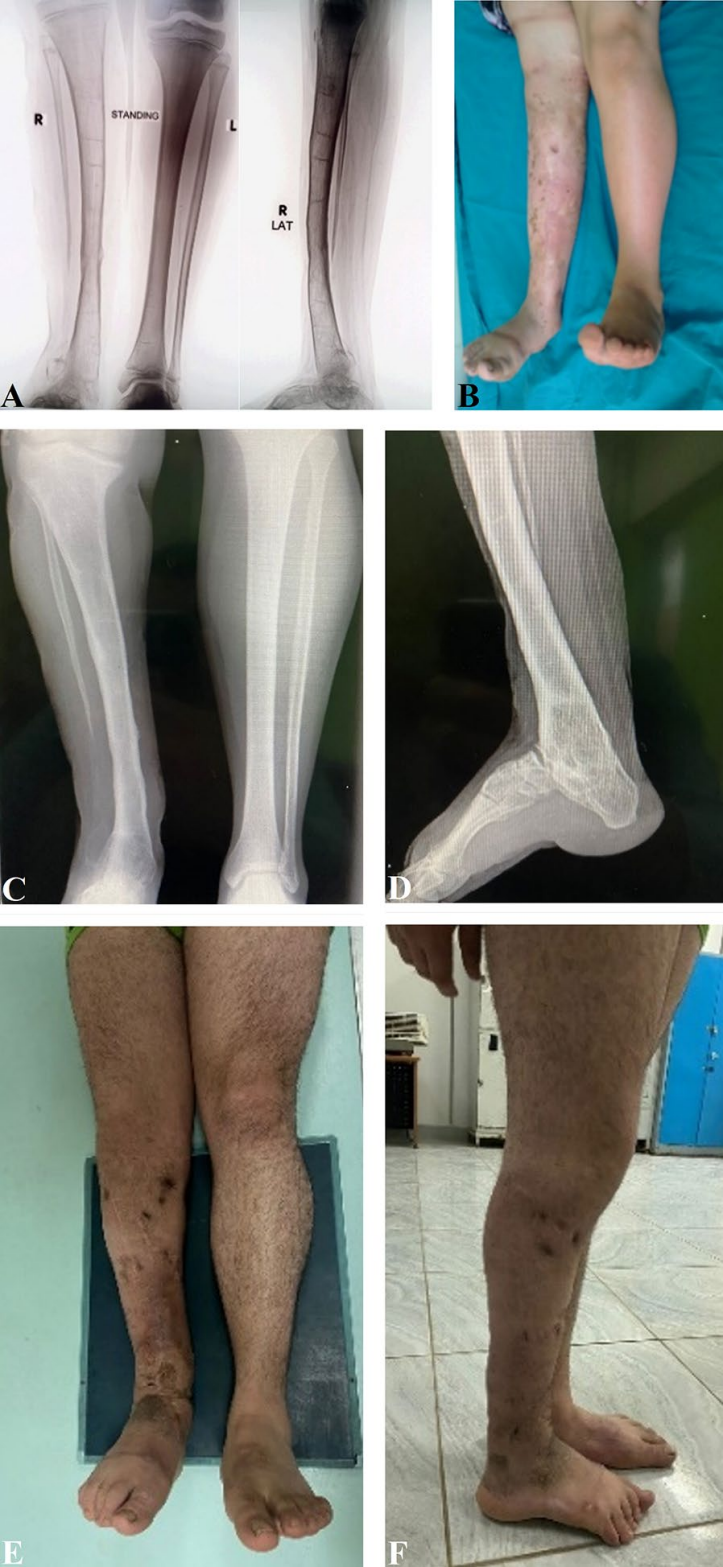
Follow-up X-rays and clinical photographs of the same patient. **A** Five years’ follow-up AP and lateral X-rays showing overgrowth of the tibia by 4 cm together with valgus ankle. **B** Five years’ follow-up clinical photographs. **C** Fifteen years’ follow-up AP view showing varus deformity at the knee with depression of the medial joint space. There is also valgus deformity at the ankle with 1.5 cm shortening. **D** Lateral X-ray of the leg and ankle shows total fusion of the ankle and subtalar joint and an increased calcaneal pitch angle. **E**, **F** Clinical photographs of the patient when he became 21 years old

Spontaneous overgrowth of the tibia regarding the length was reported in two patients at 5 and 6 years of follow-up. In one of them, this overgrowth was 4 cm at 12 years of age, but this overgrowth was reversed to 1.5 cm shortening at complete closure of the growth plates. In the second case, this overgrowth was 3 cm at the last follow-up before growth plate closure and did not resolve; see Figs. 3, 4.

Refracture occurred in two patients; in one of them, it occurred at a pin site hole, while in the other case it was at the recurrent pseudarthrosis site. In both cases, revision surgery succeeded in achieving union till late in the follow-up period (5.5 and 15 years, respectively; see Table 6).

**Table 6:** Final results and complications of surgery

No.	Follow-up (y)	LLD at final follow-up (cm)	AOFAS score at final follow-up	Major complications	Clinical results	Radiological results
1	15	− 1.5	72	Varus knee	Fair	Fair
				Valgus ankle (15 degrees)		
				Stiff fused ankle		
				Calcaneal deformity		
2	11	+ 3	87	Valgus knee	Good	Fair
				Overgrowth		
				Valgus ankle (15 degrees)		
				Procurvatum (20 degrees)		
				Decreased ankle motion		
3	2	− 10	84	Recurvatum (10 degrees)	Poor	Fair
				Decreased ankle motion		
4	5.5	− 1	96	Refracture	Excellent	Excellent
5	2	− 4	56	Non-union	Poor	Poor
				Decreased ankle motion		
6	7	− 2	75	Valgus ankle (5 degrees)	Good	Good
7	8	0	75	Valgus ankle (7 degrees)	Fair	Good
				Decreased ankle motion		
8	4	0	87	Procurvatum (7 degrees)	Good	Good
9	3	− 1	73	Decreased ankle, subtalar motion	Fair	Good
				Valgus ankle (7 degrees)		
10	6	0	75	Decreased ankle, subtalar motion	Fair	Good
				Valgus ankle (5 degrees)		
11	5	− 1	83	Valgus ankle (7 degrees)	Good	Good
				Decreased ankle motion		
12	8	0	75	Valgus ankle (12 degrees)	Fair	Fair
				Procurvatum (10 degrees)		
				Decreased ankle motion		
13	4	0	83	Valgus ankle (7 degrees)	Good	Good
14	5	− 2	84	Procurvatum (12 degrees)	Good	Fair
15	4	− 3	75	Decreased ankle, subtalar motion	Fair	Fair
				Valgus ankle (15 degrees)		
16	3	− 2	82	Valgus ankle (10 degrees)	Good	Fair

## Discussion

Congenital pseudarthrosis of the tibia (CPT) is a rare condition in children and is known for non-union and refractures. Besides achieving union and correcting deformities and limb length discrepancies (LLD), the goal of treatment is also to ensure a functional limb with minimal interventions [2–4].

In this study, we treated 16 patients with CPT with pseudarthrosis excision and the Ilizarov technique.

According to the Paley classification [7] of CPT, seven patients had type I CPT, seven had type II, and two patients had type III. However, the case with segmental tibial dysplasia could not be classified before the fracture according to this classification as there was no atrophic or sclerotic bone end and no pseudarthrosis at this stage. After the initial treatment and refracture with pseudarthrosis established, it was classified as a type I CPT. We believe that there is no single comprehensive classification that can consider all the pathological, clinical, and prognostic aspects of CPT in all circumstances.

In our study, the preoperative AOFAS score was low in most cases, with impaired limb function and an inability to bear weight on these deformed limbs with pseudarthrosis, except in one patient who was able to walk on her leg before surgery, as she only had pain because of an early stress fracture in the diseased segment, which did not advance to pseudoarthrosis till our surgery. The mean AOFAS score improved significantly from 47.5 preoperatively to 78.9 at the latest follow-up. Agashe et al. [27] treated 15 patients with CPT using a combination of the Ilizarov technique and intramedullary rodding, and the mean AOFAS score was 64 at a mean follow-up time of 4.5 years.

After the successful union of pseudarthrosis, residual long-term problems such as LLD, ankle valgus, diaphyseal malalignment, degenerative changes at the ankle joint, and calcaneus deformity were reported in patients with CPT. Proximal migration of the distal fibula accounts for progressive ankle valgus, whereas prolonged immobilization of the ankle during surgeries and later in orthosis leads to stiffness [23, 28, 29].

Tudisco et al. [10] studied ankle function in 30 patients with CPT at the end of skeletal maturity and reported that eight patients had normal ankle-joint function, 17 patients had fair to poor function, two patients underwent arthrodesis, and three patients underwent amputation.

Inan et al. [23] reported that out of 16 treated cases with CPT, the clinical results were good in 12 (75%) patients and fair in four (25%) patients, and the radiographic results were good in nine (56%) patients and fair in seven (44%) patients. In our study, based on the classification of Inan et al. [23], the clinical results were excellent in one patient, good in seven patients, fair in six patients, and poor in two patients, and the radiological results were excellent in one patient, good in seven patients, fair in seven patients, and poor in one out of the 16 treated patients.

However, we think that this classification does not consider the special nature of the disease of CPT and the great suffering of the patients and their families at the pre-union stage. In most instances, the patients were highly satisfied with just achieving permanent union of the pseudarthrosis and regaining the ability to bear weight on these long-suffering limbs, whatever the residual LLD and other deformities present (if any). However, in this grading system, > 5 cm residual shortening is considered a poor clinical result, equal to cases of persistent non-union. We think that residual shortening can be treated by limb lengthening later, while persistent CPT demands amputation. So, we believe that just achieving permanent union of the CPT should upgrade the result by one grade in any evaluation system, so that the results would be classified as fair at least.

In our study, elimination of pseudarthrosis was achieved in 15 (93.8%) cases. There was only one patient with persistent non-union and two patients with refracture. Choe et al. [20] treated 43 patients with the Ilizarov method and had refracture in 19 cases and non-union in one patient. Agashi et al. [27] treated 15 cases with the Ilizarov method and antegrade nail, and sometimes with a plate, and reported non- and delayed union in eight cases and refracture in one case and recommended amputation in one case. Mathieu et al. [14] treated 10 patients with the Ilizarov method and antegrade or retrograde nailing and followed up for 4 years, and noted two cases of non-union and one case of refracture.

Different surgical procedures can be used alone or in combination to treat CPT, such as intramedullary rods, vascularized fibular grafting, the Ilizarov technique, or the cross-union method. Successful surgery should achieve total excision of the lesion, deformity correction, bone healing promotion, and limb lengthening [3, 12–14].

Ilizarov ring fixation with lengthening or bone transport has proven to be the most effective treatment method [30]. The intramedullary nailing method includes resection of the pseudarthrosis, tibial shortening, fixation with an intramedullary rod, and bone grafting [31]. Free vascularized fibular grafting is a reliable technique for consolidation but has the complication of donor site morbidity [13]. The cross-union method involves intentional cross-union between the tibia and fibula [3]. Some studies have reported better union and fewer refracture rates when using this method [3, 32]. However, we think that the cross-union technique has a limitation in patients with preoperative atrophic fibula combined with pseudarthrosis. Also, the most common problem observed following cross-union has been telescopic nail pull-out of the proximal or distal epiphysis, which can be remedied by a hemi-epiphysiodesis plate and exchange rod [33].

CPT remains a true challenge, not only in terms of achieving union and correcting associated deformities, but also in terms of anticipating, predicting, and treating late-onset postoperative residual complications after the complete union of CPT. To decrease non-union and refracture rates, we recommend keeping the cross-sectional area at the docking site as wide as possible and avoiding premature removal of the fixator. To achieve the best ankle function and decrease the incidence of stiffness in the ankle and hindfoot, we recommend conversion to intramedullary fixation after the complete union of CPT in order to discard the orthosis and allow joint motion as early as possible.

This study has some limitations, including the non-routine use of MRI, which could be useful in evaluating fibrous hamartomas, periosteum, and bone lesions preoperatively to better identify the extent of the soft tissue and bone lesions to be excised. Other limitations include its retrospective nature, relatively small number of cases, the use of an internal intramedullary rod following fixator removal in only some of the patients, and the fact that only two patients reached full skeletal growth at the end of follow-up.

## Conclusion

Congenital pseudarthrosis of the tibia can be adequately managed by the Ilizarov method, which may be combined with an intramedullary rod and a primary or secondary bone graft, with good long-term functional and radiological outcomes. This method has a high union rate and can provide simultaneous effective management of problems with pseudarthrosis, including non-union, deformity, limb shortening, and adjacent joint contracture and subluxation.

## Data Availability

The dataset analyzed in this study is available from the corresponding author on request.
